# Preoperative proteinuria is associated with increased rates of acute kidney injury after partial nephrectomy

**DOI:** 10.1590/S1677-5538.IBJU.2018.0776

**Published:** 2019-01-29

**Authors:** Önder Kara, Matthew J. Maurice, Pascal Mouracade, Ercan Malkoc, Julien Dagenais, Mustafa Çapraz, Jaya S. Chavali, Merve Yazici Kara, Jihad H. Kaouk

**Affiliations:** 1 Department of Urology Glickman Urological and Kidney Institute Cleveland Clinic Cleveland OH USA Department of Urology, Glickman Urological and Kidney Institute, Cleveland Clinic, Cleveland, OH, USA;; 2 Kocaeli University Medical School Kocaeli Turkey Kocaeli University, Medical School, Kocaeli, Turkey;; 3 Amasya University Medical School Amasya Turkey Amasya University, Medical School, Amasya, Turkey;; 4 Kocaeli Derince Training and Research Hospital Kocaeli Turkey Kocaeli Derince Training and Research Hospital, Kocaeli, Turkey

**Keywords:** Kidney Neoplasms, Proteinuria, Acute Kidney Injury

## Abstract

**Purpose:**

We investigated the association between preoperative proteinuria and early postoperative renal function after robotic partial nephrectomy (RPN).

**Patients and Methods:**

We retrospectively reviewed 1121 consecutive RPN cases at a single academic center from 2006 to 2016. Patients without pre-existing CKD (eGFR≥60 mL/min/1.73m^2^) who had a urinalysis within 1-month prior to RPN were included. The cohort was categorized by the presence or absence of preoperative proteinuria (trace or greater (≥1+) urine dipstick), and groups were compared in terms of clinical and functional outcomes. The incidence of acute kidney injury (AKI) was assessed using RIFLE criteria. Univariate and multivariable models were used to identify factors associated with postoperative AKI.

**Results:**

Of 947 patients, 97 (10.5%) had preoperative proteinuria. Characteristics associated with preoperative proteinuria included non-white race (p<0.01), preoperative diabetes (p<0.01) and hypertension (HTN) (p<0.01), higher ASA (p<0.01), higher BMI (p<0.01), and higher Charlson score (p<0.01). The incidence of AKI was higher in patients with preoperative proteinuria (10.3% vs. 4.6%, p=0.01). The median eGFR preservation measured within one month after surgery was lower (83.6% vs. 91%, p=0.04) in those with proteinuria; however, there were no significant differences by 3 months after surgery or last follow-up visit. Independent predictors of AKI were high BMI (p<0.01), longer ischemia time (p<0.01), and preoperative proteinuria (p=0.04).

**Conclusion:**

Preoperative proteinuria by urine dipstick is an independent predictor of postoperative AKI after RPN. This test may be used to identify patients, especially those without overt CKD, who are at increased risk for developing AKI after RPN.

## INTRODUCTION

Partial nephrectomy (PN) is the gold standard treatment for T1a, and when technically feasible T1b renal masses, due to improved renal functional preservation (1). Given the benefits offered by the robotic platform, there has been an upward trend in the utilization of robotic partial nephrectomy (RPN) for the treatment of renal masses, and relatedly robotic adoption has increased the overall utilization of nephron-sparing surgery (2, 3). PN reduces the amount of renal parenchymal volume loss, more so when performed robotically; however; it does not eliminate nephron loss entirely (4-6). Furthermore, the remaining kidney may experience ischemic damage as a result of the temporary vascular clamping required during PN (5). Acute kidney injury (AKI) is associated with increased hospital length of stay and in hospital mortality (7), and following AKI patients have an increased risk of residual structural and functional disease (8). Therefore, preoperative prediction of AKI, especially for patients with presumed normal renal function (Estimated Glomerular Filtration Rate (eGFR)>60) is difficult, and important in patients’ counseling.

The glomerular filtration rate (GFR) has been used for a long time as the primary indicator in diagnosing and staging CKD (9). However, Kidney Disease Improving Global Outcomes (KDIGO) recently included etiology, eGFR, and proteinuria as vital components for CKD identification, as each of them has a prognostic value on survival and renal function stability in the population (10-12). The association between CKD severity and AKI risk after PN was quantified based on one component of KDIGO classification (as measured by levels of estimated GFR) in previously published series (13-15). Our primary objective was to assess proteinuria as a marker of early renal dysfunction in patients undergoing RPN.

## PATIENTS AND METHODS

Using our institutional review board-approved database, we abstracted data on 1121 RPN cases performed at our center from 2006 to 2016. Patients without pre-existing CKD (eGFR≥60 mL/min/1.73m^2^) who had a urinalysis within 1-month prior to RPN were included in the study (n=947). The cohort was categorized by the presence or absence of preoperative proteinuria, and groups were compared in terms of clinical and functional outcomes. The incidence of AKI was assessed using RIFLE criteria. Univariate and multivariable models were used to identify factors associated with postoperative AKI.

### Definition of proteinuria

Urine dipstick analysis was used to detect proteinuria. Proteinuria was defined as presence (trace or greater (≥1+) urine dipstick), and absence (negative urine dipstick).

### Definition of AKI

The diagnosis of AKI was based on RIFLE criteria (16). Grade 1 (risk) is characterized by a 1.5-2.0-fold increase in serum creatinine or urine output (UO)<0.5mL/kg/h for 6 hours; grade 2 (injury) is characterized by a 2.0-3.0-fold increase in serum creatinine or UO 0.5mL/kg/h for 12 hours; grade 3 (failure) is characterized by any increase >3.0-fold in serum creatinine, temporary need for dialysis, UO <0.3mL/kg/h for 12 hours, or anuria for 12 hours. There were no cases of renal loss or end-stage renal failure in this cohort of patients.

### Study variables

Demographic and tumor characteristics included patient age; race (white and non-white); gender; body mass index (BMI); Charlson Comorbidity Index (CCI); American Society of Anesthesiology (ASA) score; history of preoperative diabetes mellitus (DM), hypertension (HTN), and/or smoking; preoperative estimated glomerular filtration rate (eGFR); solitary kidney status; tumor size; R.E.N.A.L. score; and tumor pathology (benign or malignant). Intraoperative variables included operative time, ischemia time, estimated blood loss (EBL), and intraoperative transfusion. Postoperative variables included 30-day postoperative complications, length of hospital stay, and 30-day readmissions. Complications were graded using the Clavien-Dindo classification system (17) and were characterized as minor (Clavien 1-2) and major (Clavien 3-5). Tumor complexity was assessed based on the R.E.N.A.L nephrometry classification system (18). Functional outcomes were assessed using eGFR, which was calculated using the modification of diet in renal disease (MDRD) formula (19). eGFR preservation was defined as follow-up postoperative eGFR divided by baseline eGFR x 100. CKD upstaging was defined as any increase in CKD stage (20) from the time of preoperative assessment to the time of latest postoperative follow-up.

### Surgical technique

We used our standard RPN technique as described previously (21). The transperitoneal approach was used in all cases. Intraoperative ultrasound was used routinely for intraoperative tumor identification and surgical planning. Intracorporeal renal parenchymal cooling was used selectively when ischemia times were expected to be greater than 25 minutes.

### Study outcomes

The primary outcome was postoperative AKI. AKI was assessed using RIFLE criteria. Univariate and multivariable models were used to identify factors associated with postoperative AKI. Secondary outcomes included operative time, EBL, ischemia time, perioperative transfusion, length of hospital stay, 30-day readmission, overall and major complications.

### Statistical analysis

Continuous variables, presented as mean ± standard deviation (SD) if normally distributed or as median (interquartile range (IQR)) if non-normally distributed, were compared using the t-test or Mann-Whitney U test, respectively. Categorical variables were compared using the chi-squared test. Multivariable analysis was conducted using logistic regression to identify independent predictors of postoperative AKI. Significance was set at p<0.05. Analyses were performed using SPSS v24 software (IBM SPSS Statistics, Armonk, NY: IBM Corp).

## RESULTS

In the final cohort, 947 patients were included. Preoperative proteinuria was observed in 97 (10.5%) patients on urine dipstick. Of these, 18 (18.5%) had trace (<30 mg/dL), 78 (80.4%) had 30 to 299 mg/dL, and 1 (1.1%) had >300 mg/dL urinary protein preoperatively. Characteristics associated with preoperative proteinuria included non-white race (p<0.01), pre-existing DM (p<0.01), pre-existing HTN (p<0.01), higher BMI (p<0.01), higher ASA (p<0.01), and higher Charlson score (p<0.01). Tumor characteristics, including mass size (p=0.08), R.E.N.A.L score (p=0.13), and malignant disease (p=0.06), were not associated with preoperative proteinuria (Table-1).

**Table 1:** Patient demographic and tumor characteristics.

Variables	Proteinuria		P value
	Yes N=97 (10.5%)	No N=850 (89.5%)	
Age years, (±SD)	57.1 (12.7)	57.7 (12)	0.79
**Gender**			**0.11**
Male, n (%)	65 (67)	499 (58.7)	
Female, n (%)	32 (33)	351 (41.3)	
**Race**			**<0.01**
White, n (%)	75 (77.3)	756 (89)	
Non-White, n (%)	22 (22.7)	144 (11)	
BMI, med (IQR)	31.2 (26.2-37)	29.3 (25.8-33.5)	**0.04**
ASA, med (IQR)	3 (3-3)	3 (2-3)	**<0.01**
CCI, med (IQR)	1 (0-2)	0 (0-1)	**<0.01**
Diabetes Mellitus, n (%)	29 (29.9)	154 (18.1)	**<0.01**
Hypertension, n (%)	64 (66)	451 (53.1)	**0.01**
Smoker, n (%)	13 (13.4)	123 (14.5)	0.77
Pre-Op eGFR, med (IQR)	87.2 (71.3-102.2)	88.5 (76–100.5)	0.58
Solitary kidney, n (%)	3 (3.1)	13 (1.5)	0.22
Tumor size on CT cm, med (IQR)	3.4 (2.2-4.4)	3 (2.1-4)	0.08
R.E.N.A.L score, med (IQR)	8 (6–9)	7 (6–9)	0.13
Malignant disease, n (%)	86 (88.7)	682 (80.2)	0.06

**BMI** = Body mass index; **CCI** = Charlson comorbidity index; **CKD** = Chronic kidney disease; **CT** = Computed tomography; **EBL** = Estimated blood loss; **eGFR** = Estimated glomerular filtration rate; **IQR** = Interquartile range; **OPN** = Open partial nephrectomy; **RPN** = Robotic partial nephrectomy; **SD** = Standard deviation

Postoperative AKI was more prevalent in patients with preoperative proteinuria (10.3% vs. 4.6%, p=0.01). The median eGFR preservation measured within one month after surgery was lower (83.6 (73.3-89.8) % vs. 91 (79-101) %, p=0.04) in those with proteinuria; however, there were no significant differences by 3 months after surgery (88 (77.3-98.4) % vs. 89 (78.2-97.5) %, p=0.9) or last follow-up visit (85.1 (72.9-96.2) % vs. 86.9 (76.1–98.2) %, p=0.2). Likewise, the prevalence of CKD upstaging at the latest follow-up (19.5 vs. 18.7 months, p=0.56) did not differ between groups (43.3% vs. 42.1%, p=0.82) (Table-2).

**Table 2:** Follow-up functional data.

Variables	Proteinuria		P value
	Yes N=97 (10.5%)	No N=850 (89.5%)	
**Early postoperative functional outcomes (Primary Outcomes)**			
**Acute kidney injury (RIFLE), n (%)**	1**0 (10.3)**	**39 (4.6)**	**0.01**
Risk (R)	9 (9.3)	35 (4.1)	
Injury (I)	1(1)	4 (0.5)	
Within 1 mo. eGFR, mL/min/1.73 m^2^, median (IQR)	73 (63-90)	80 (69-98)	**0.02**
Within 1 mo. % eGFR preservation, median (IQR)	83.6 (73.3-89.8)	91 (79-101)	**0.04**
**Late postoperative functional outcomes**			
3-mo. eGFR, mL/min/1.73 m^2^, median (IQR)	76 (61-94.9)	77 (65.3-91.8)	0.98
3-mo. % eGFR preservation, median (IQR)	88 (77.3-98.4)	89 (78.2-97.5)	0.9
Follow up times, months, median (IQR)	19.5 (6.2-29.4)	18.7 (5.7-38.4)	0.56
Latest eGFR, mL/min/1.73 m^2^, median (IQR)	72 (61.1–89.1)	76 (64.3–90.4)	0.12
Latest follow up % eGFR preservation, median (IQR)	85.1 (72.9–96.2)	86.9 (76.1–98.2)	0.2
CKD upstaging at last follow-up, n (%)	42 (43.3)	358 (42.1)	0.82

**IQR** = Interquartile range; **SD** = Standard deviation

In terms of secondary outcomes, there were no significant differences in intraoperative variables, including operative time, EBL, ischemia time, and intraoperative blood transfusion between the two groups. However, proteinuria was associated with higher rates of overall (26.8% vs. 16.8, p=0.01) and major (9.3% vs. 4.6%, p=0.04) postoperative complications and 30-day readmissions (Table-3).

**Table 3:** Secondary outcomes.

Variables		Proteinuria			P value	
		Yes N=97 (10.5%)		No N=850 (89.5%)		
**Intraoperative outcomes**						
Operation time, min, mean (±SD)	182 (48.8)		180 (53)			0.34
EBL, mL., med (IQR)	150 (100-300)		150 (100-250)			0.92
Ischemia time, min, mean (±SD)	20.8 (10)		20.3 (10.1)			0.68
Intraoperative transfusion, n (%)	2 (2.1)		7 (0.8)			0.23
**Perioperative outcomes**						
Length of stay, days, med, (IQR)		3 (2-4)		3 (2-4)	0.23	
30-day readmission, n (%)		8 (8.2)		31 (3.6)	**0.03**	
Postoperative transfusion, n (%)		2 (2.2)		48 (5.9)	0.22	
Overall C. (Clavien-Dindo 1-5), n (%)		26 (26.8)		143 (16.8)	**0.01**	
Major C. (Clavien-Dindo 3-5), n (%)		9 (9.3)		39 (4.6)	**0.04**	

On further analysis of postoperative complications, the specific complications, which contributed to the disparity in complication rates between groups, included postoperative cardiac complications (7.2% vs. 2.6, p=0.02), and haemorrhagic complications necessitating selective arterial angioembolisation (4.1% vs. 0.9%, p=0.02) (Table-4).

**Table 4:** Summary of complications based on preoperative proteinuria.

	Proteinuria		P value
Complication type, % (n)	Yes N=97 (10.5%)	No N=850 (89.5%)	
**Overall complications**	**26 (26.8)**	**143 (16.8)**	**0.01**
Major (Clavien-Dindo 3-5)	9 (9.3)	39 (4.6)	**0.04**
**Cardiac complications**	**7 (7.2)**	**22 (2.6)**	**0.02**
Myocardial infarction	1 (1)	0 (0)	
Arrhythmia	4 (4.1)	16 (1.9)	
Other cardiac	2 (2.1)	6 (0.7)	
**Pulmonary complications**	**4 (4.1)**	**51 (6)**	**0.64**
Pneumonia	1 (1)	9 (1.1)	
DVT/PE	1 (1)	11 (1.3)	
Other pulmonary	2 (2.1)	31 (3.6)	
**Genitourinary complications**	**3 (3.1)**	**13 (1.5)**	**0.22**
UTI	2 (2.1)	4 (0.5)	
Urine leak	1 (1)	9 (1.1)	
**Gastrointestinal complications**	**5 (5.2)**	**32 (3.8)**	**0.41**
Clostridium difficil infection	1 (1)	1 (0.1)	
Ileus/small bowel obstruction	3 (3.1)	29 (3.4)	
Other gastrointestinal	1 (1)	2 (0.2)	
**Wound complications**	**2 (2.1)**	**18 (2.1)**	**1**
Surgical site infection	0 (0)	13 (1.5)	
Hernia	0	3 (0.4)	
Other wound	2 (2.1)	2 (0.2)	
Neurologic complications	0	2 (0.2)	1
Bleeding complications	9 (6.3)	11 (14.1)	
Postop Transfusion	2 (2.1)	48 (5.6)	0.22
Need for angioembolisation	4 (4.1)	8 (0.9)	**0.02**

On multivariable logistic regression, after adjusting for BMI, CCI, preoperative proteinuria, tumor size, baseline eGFR, and ischemia time, significant predictors of postoperative AKI included higher BMI (OR 1.07, 95% CI 1.03-1.17, p<0.01), ischemia time >20 min (OR 4.86, 95% CI 2.14-11.01) p<0.01), and preoperative proteinuria (OR 2.4, 95% CI 1.02-5.65, p=0.04) (Table-5).

**Table 5:** Logistic regression analysis predicting AKI after PN.

Variables	Univariate			Multivariate		
	OR	95 % CI	P	OR	95 % CI	P
Age (per year)	0.99	0.97-1.01	0.3			
**Race**						
(White vs. Non-White)	0.66	0.3-1.45	0.4			
Gender (Male vs. Female	1.01	0.56-1.82	0.95			
BMI (per kg/m^2^)	1.07	1.04-1.11	**<0.01**	1.07	1.03-1.11	**<0.01**
CCI (per unit)	1.21	0.98-1.49	**0.01**	1.19	0.95-1.48	0.12
Hypertension (yes vs.no)	1.34	0.74-2.42	0.32			
Diabetes (yes vs.no)	1.37	0.7-2.7	0.34			
Proteinuria (yes vs. no)	2.3	1.15-4.95	**0.01**	2.4	1.02-5.65	**0.04**
Tumor size (per cm)	1.28	1.11-1.47	**<0.01**	1.05	0.87-1.27	0.55
Baseline eGFR (per mL/min/1.73m^2^)	1.01	0.99-1.02	0.11	1.01	0.99-1.02	0.07
Ischemia time ≤20 min.	Ref			Ref		
Ischemia time >20 min	4.63	2.13-10.4	**<0.01**	4.86	2.14-11.01	**<0.001**
EBL (per cc)	1	1-1	0.38			
IVF during surgery	1	1-1	0.57			

**CI** = Confidential interval; **EBL** = Estimated Blood Loss; **OR** = Odds ratio

## DISCUSSION

Despite the nephron-sparing benefits of PN, 4.9% of patients undergoing PN experience postoperative AKI (15). In turn, AKI is associated with increased morbidity and mortality (22). While preexisting CKD is one of the most common risk factor for postoperative AKI after PN, even patients with normal preoperative renal function are at risk for postoperative AKI (23). However, at present, these at-risk patients without CKD are not readily identifiable. Thus, there is a need for better tools to identify such patients who are more likely to experience AKI after PN.

Proteinuria has been identified as an essential component of renal dysfunction based on the most recent KDIGO guidelines (12) and appears to be a significant and independent predictor of overall survival and recurrence free survival in patients undergoing renal cancer surgery (24). Therefore, we hypothesized that preoperative proteinuria may be associated with postoperative AKI.

In this retrospective study, the prevalence of postoperative AKI was 5.1%. Some studies have reported postoperative AKI rates after PN ranging from 0.8% to 10% (13, 15, 25) varying by institution, technique, approach, data collection, and AKI criteria. In our study, we used the RIFLE classification scheme for AKI, which is generally accepted for use in the PN population (24).

We found that proteinuria was an independent risk factor for AKI in non-CKD patients undergoing PN. Patients with proteinuria had 2.4-fold higher odds of AKI than patients without proteinuria. These results are consistent with prior studies that have shown an association between proteinuria and AKI after non-renal (26-28), and renal surgeries (29). Surprisingly, in our study, preoperative proteinuria was not a predictor of long-term renal functional preservation. This finding contrasts a study by Krane et al. (30) and Bhindi et al. (29), and O’Donnell et al. (31) which did show an association between proteinuria and long-term risk of CKD. It is possible that our follow up was not long enough to detect a difference in long-term functional outcomes.

Our findings suggest that proteinuria detected on urine dipstick is a good predictor of postoperative AKI in non-CKD patients. Urine dipstick is quick, inexpensive, and widely available, making it a good screening test. Preoperative assessment of proteinuria may help guide preoperative patient counseling, postoperative care, and medical treatment in non-CKD PN patients.

In addition to proteinuria, longer ischemia time and higher BMI were also independent risk factors for AKI. Our study demonstrated a 4.8-fold higher risk of AKI in patients with ischemia times longer than 20 minutes. The association between longer ischemia time and increased risk of post-PN AKI is well established in the literature (32-34). In terms of patient factors, BMI was the only independent predictor of post-PN AKI. Obesity has been identified previously as a risk factor for AKI after surgery, consistent with our results (35). The pathophysiology of obesity-associated AKI is poorly understood but may be related to comorbidities, such as DM and HTN.

Our study suggests an increased risk of overall and major complications and 30-day readmissions following PN in patients with proteinuria. This association did not persist on multivariable logistic regression analysis, suggesting that comorbid conditions, which occur commonly together with proteinuria, may be responsible for this increased morbidity. Specifically, postoperative cardiovascular complications were more common in patients with proteinuria, consistent with prior studies that have shown an association between proteinuria and cardiovascular morbidity and mortality across divergent populations (36).

Our study is not without limitations. The retrospective design is a potential source of bias, and results from this single tertiary-care center cohort may not be generalizable. While multivariable analysis was used to adjust for known risk factors for postoperative AKI, additional unmeasured factors, for which we could not adjust, may have influenced the ultimate risk of AKI. Another limitation is that urine dipstick was used rather than 24-hour urinalysis for the assessment of proteinuria. Although a 24-hour urinalysis would be the ideal study for proteinuria, it is a more expensive and cumbersome test that would not be practical in all patients undergoing PN.

## CONCLUSIONS

Our results indicate that preoperative proteinuria by urine dipstick is an independent predictor of postoperative AKI after RPN in non-CKD patients. This test may be used to identify patients with occult renal dysfunction who are at increased risk for developing post-PN AKI.

### Compliance with Ethical Standards

Dr. Jihad H. Kaouk is a consultant for Endocare/HealthTronics, and Intuitive. No competing financial interests exist for the other authors.
